# Real-time monitoring of cell protrusion dynamics by impedance responses

**DOI:** 10.1038/srep10206

**Published:** 2015-05-15

**Authors:** Paolo Armando Gagliardi, Alberto Puliafito, Laura di Blasio, Federica Chianale, Desiana Somale, Giorgio Seano, Federico Bussolino, Luca Primo

**Affiliations:** 1 Department of Oncology, University of Torino, Torino, 10043, Italy; 2 Candiolo Cancer Institute-FPO IRCCS, Candiolo, 10060, Italy; 3 Center for Molecular Systems Biology, University of Torino, 10124, Torino, Italy

## Abstract

Cellular protrusions are highly dynamic structures involved in fundamental processes, including cell migration and invasion. For a cell to migrate, its leading edge must form protrusions, and then adhere or retract. The spatial and temporal coordination of protrusions and retraction is yet to be fully understood. The study of protrusion dynamics mainly relies on live-microscopy often coupled to fluorescent labeling. Here we report the use of an alternative, label-free, quantitative and rapid assay to analyze protrusion dynamics in a cell population based on the real-time recording of cell activity by means of electronic sensors. Cells are seeded on a plate covered with electrodes and their shape changes map into measured impedance variations. Upon growth factor stimulation the impedance increases due to protrusive activity and decreases following retraction. Compared to microscopy-based methods, impedance measurements are suitable to high-throughput studies on different cell lines, growth factors and chemical compounds. We present data indicating that this assay lends itself to dissect the biochemical signaling pathways controlling adhesive protrusions. Indeed, we show that the protrusion phase is sustained by actin polymerization, directly driven by growth factor stimulation. Contraction instead mainly relies on myosin action, pointing at a pivotal role of myosin in lamellipodia retraction.

Cell migration plays crucial roles in many physiological processes and contributes to cancer cells invasion and dissemination. Migration strategies employed by cells change in response to the diverse environmental stimuli, such as rigidity of the substrate, molecular composition of the extracellular matrix or spatio-temporally varying concentrations of soluble molecules such as growth factors or cytokines. Typically, migration through/on a matrix involves the generation of cell protrusions, i.e. extensions of plasma membrane outside the cell body^1^. So far, different types of protrusion have been identified to contribute to cell migration and invasion in specific contexts, cell types and microenvironment^2^. For example, fibroblasts form either lamellipodia^3^ or lobopodia^4^ according to extracellular matrix dimensionality and elasticity. Filopodia are more explorative structures^5^ and are relevant in the guidance of neuronal growth cones^6^ and endothelial tip cell during sprouting angiogenesis^7^. Membrane blebs instead are typical of amoeboid type of cell migration and invasion and have been described in leucocytes^8^, D. discoideum^9^ and H. histolytica^10^. In lamellipodia and filopodia actin polymerization drives forward protrusion of the plasma membrane^2^. For this reason, much emphasis has been placed on delineating molecular regulators and upstream cellular signaling of actin polymerization, which in turn control cell protrusion formation^11^. However, the dynamics of cell protrusions also include their retraction. Extension and retraction should occur in a coordinated fashion in order to drive efficient cell migration^12^.

A challenging feature of studying protrusion dynamics is the ability to provide quantitative as well as time-resolved data. The most common approach to this problem is the use of live-microscopy on 2D adherent cells which employs different imaging techniques such as standard wide-field, confocal or total internal reflection fluorescence (TIRF) microscopy^13^,^14^,^15^. There exist advanced implementations of these methods such as Stroboscopic Analysis of Cell Dynamics^16^ and fluorescent speckle microscopy, which visualizes the movement and assembly/disassembly of actin filaments in protrusive structures^17^. Atomic force microscopy has also been used to measure lamellipodia dynamics and thickness in adenocarcinoma cells or in migrating keratocytes^18^,^19^.

These approaches are powerful as they all allow single cell or even subcellular resolution, and represent the method of choice to study protrusion dynamics. However, such methods present a few drawbacks: i) they often require complex image and/or mathematical processing to obtain quantitative results, ii) they are hardly suitable for high throughput studies such as biochemical functional or drug screening and iii) are subject to cell to cell variability.

Here, we make use of a well-established technique based on the measurement of the frequency dependent electrical impedance of cell-covered electrodes subject to a small alternate electric current^20^,^21^. Cells adhering on the electrodes vary the impedance in a frequency dependent manner. By properly modulating the frequency of the current, its amplitude, the time duration of the experiment and the size and arrangement of electrodes, a number of different biological processes can be quantified^21^,^22^,^23^,^24^,^25^,^26^,^27^.

Here we employ the impedance reading (IR) technique to quantitatively measure protrusion dynamics and validate the results by direct comparison with quantitative data of cell surface variation, obtained through image analysis of live TIRF microscopy. Our data provide insights on how lamellipodia protrusion and retraction are regulated. We present data directly and quantitatively linking the amplitude of the response and its kinetics to the ligand concentration. By direct comparison of microscopy data with IR data we can dissect the different parts of the response into molecularly distinct events mediated by actin polymerization and myosin contraction that can be inhibited separately by specific drugs.

## Results

### Impedance reading variations can be quantitatively mapped into cell protrusion dynamics

**Figure 1:**
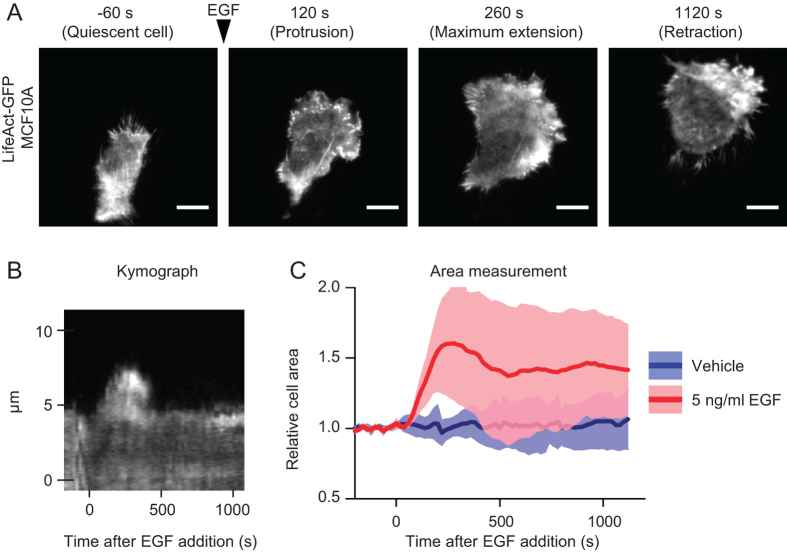
EGF stimulation induces protrusion formation and retraction. (**A**) MCF10A cells were infected with pLKO.1 LifeAct-GFP, deprived of EGF for 6 hours and kept in a humidified chamber at 37 °C and 5% CO2. Cells were then imaged by means of TIRF microscopy. The depth of the evanescent field was kept at 90 nm. Cells were imaged over a time period of 1180 seconds and either stimulated or not with 5 ng/ml EGF. The time at which the stimulus was added was set to t = 0 seconds. Images were acquired every 20 seconds (Video S1). Four time-points are reported in the figure: −60 seconds (before EGF addition, quiescent cell), 120 seconds (protrusion), 260 seconds (maximum extension) and 1120 seconds (retraction). Scale bars, 10 μm. (**B**) Kymograph of a pLKO.1 LifeAct-GFP MCF10A cell stimulated as in A. A segment perpendicular to lamellipodium was used to monitor fluorescence intensity at each time points, and then the different time points were assembled. (**C**) Cell surface area variation observed by TIRF microscopy of pLKO.1 LifeAct-GFP MCF10A cells stimulated with EGF or unstimulated in which only the medium containing EGF was added (vehicle). Dark thick lines represent the mean values while light shades represent the area included between + SD and – SD.

The non-transformed mammary epithelial cells MCF10A are highly sensitive to EGF stimulation in chemotaxis^28^ and, when sparsely seeded on a flat substrate, respond to EGF stimulation by producing fast growing lamellipodia^29^. We investigated protrusive activity in MCF10A cells by imaging cells stably transduced with LifeAct-GFP by TIRF microscopy. Under these experimental conditions, lamellipodia were easily identifiable as rapid growing flat meshes of actin filaments^1^. Upon EGF stimulation, cells extended lamellipodia for about 100–400 seconds, and started then to retract, slowly reducing the adhering surface (Fig.1A, Video S1). This behavior is clearly visible in a kymograph representation (Fig. 1B). To quantitatively measure protrusion dynamics we calculated in each time frame the area of cells adherent to the surface. While the area was stable in untreated cells, EGF stimulation caused a rapid increase of cell area that, after reaching the maximum value, decreased (Fig. 1C).

This approach is powerful and quantitative but it is time expensive and biased by cell to cell variability. In order to be able to observe protrusion dynamics in a more rapid and reproducible manner, we employed a method based on IR. To quantify the impedance change over-time, a unit-less parameter is used that evaluates the relative change of impedance with respect to a reference impedance value without cells and to the impedance at beginning of the measurement. This parameter is called the cell index (CI). An increase in cell surface area contacting the electrodes will result in a larger impedance and consequently in an increase in cell index. Conversely, when cell diminish their adherent surface area, the cell-index will decrease. To better compare IR experimental measurements obtained under unstimulated and stimulated conditions, cell-index is further post-processed to obtain a quantity called Baseline Δ Cell Index. Further details are given in the Materials and Methods section.

**Figure 2:**
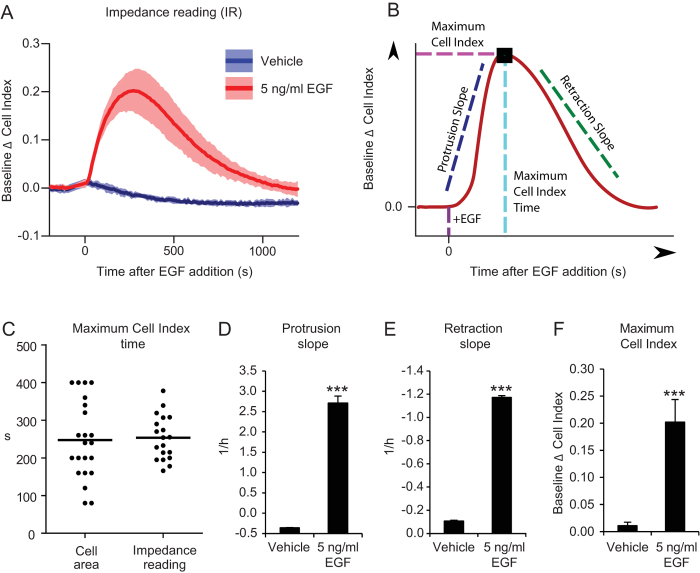
Real-time evaluation of cell protrusion dynamics by IR. (**A**) Baseline Δ cell index values obtained by IR of MCF10A cells stimulated or not with EGF at t = 0. (**B**) Cartoon showing the indicators that we use for quantification of the IR response to EGF stimulation. The point where the EGF response curve reaches the highest value corresponds to Maximum Cell Index and the Maximum Cell Index Time (t^m^). The protrusion slope is calculated as the mean slope between t^0^ and t^m^, where t^0^ is the first time point after growth factor addition. The retraction slope is calculated as the mean slope between t^m^ and t^2m^, where t^2m^ is twice t^m^. (**C**) Maximum Cell Index Time (t^m^) of a number of experiments performed by means of TIRF microscopy or IR of MCF10A stimulates with 10 ng/ml of EGF. Each point represents a separate experiment. Dashes represent the mean values. (**D**) Protrusion slope, (**E**) retraction slope and (**F**) maximum value of Baseline Δ cell index curve of MCF10A stimulated or not with 5 ng/ml EGF. ***P < 0.001.

MCF10A cells generated a reproducible and specific impedance variation pattern in response to EGF stimulation: immediately after EGF addition CI increased rapidly, reaching a maximum between 100 and 400 seconds, and then decreasing back close to initial values (Fig. 2A). In order to precisely compare IR and TIRF microscopy data we defined 4 quantitative indicators, schematically explained in Fig. 2B: the time at which the cell-index reaches its maximum indicating how fast is the response globally, the value of cell-index at maximum indicating the intensity of the response, and protrusion or retraction slopes indicating the steepness of the protrusion or retraction phases respectively (Fig. 2B).

Both IR and TIRF microscopy approaches revealed that the mean maximum cell index time was at about 250 seconds (Fig. 2C), thus indicating that IR is indeed a reliable readout for adherent surface area. Furthermore, protrusion and retraction slopes and the maximum intensity of response (maximum CI) were all significantly modulated by EGF stimulation and allow quantitative comparison between different experiments (Fig. 2D,E,F).

### EGF stimulation affects cell protrusion dynamics in a concentration dependent manner

To assess the reliability of IR in detecting cell protrusion dynamics we first performed a control experiment to establish the linearity of the CI with respect to cell density. By increasing the number of plated cells, the shape of the curves was similar while the maximum value of cell index raised (Fig. S1A). In particular we found that the maximum cell index values of EGF-stimulated curves increased linearly with the cell number in the range between 1 × 10^3^ and 3 × 10^4^ cells (Fig. S1C). Cell densities lower than 3 × 10^3^ produced maximum cell index values upon EGF stimulation almost undistinguishable from that of vehicle treated cells (Fig. S1D). Therefore, we used 5 × 10^3^ cells/well in all experiments.

To become sensitive to EGF stimulation cells need a period of EGF deprivation. We investigated how the length of EGF deprivation influenced the protrusion dynamics (Fig. S1B) and we observed that short EGF deprivation time (0.5 hours) was sufficient to detect protrusion and contraction in response to EGF addition. However, better responses in term of maximum cell index were achieved after an EGF deprivation period ranging between 2 and 8 hours (Fig. S1E). Interestingly, longer EGF deprivation times reduced the cell ability to produce protrusions in response to EGF (Fig. S1E). Therefore we used in following experiments a starving period of 6 hours.

**Figure 3:**
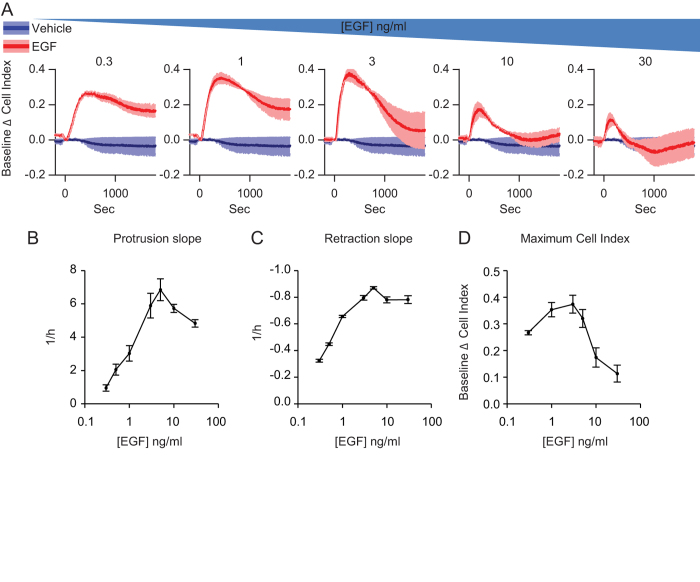
EGF affects protrusion dynamics in a concentration dependent manner. 5 × 10^3^ MCF10A cells were deprived of EGF for 6 hours and stimulated with increasing concentrations of EGF. (**A**) IR of MCF10A cells stimulated with 0.3, 1, 3, 10, 30 ng/ml of EGF. (**B**) Protrusion slope, (**C**) retraction slope and (**D**) maximum value of Baseline Δ cell index in function of EGF concentration.

Then, we evaluated the effect of varying concentration of EGF on protrusion dynamics by employing 0.3 ng/ml to 30 ng/ml (Fig. 3A). Even at 0.3 ng/ml we were able to detect variations of impedance values. Interestingly, we observed that protrusion slope (Fig. 3B), retraction slope (Fig. 3C) and maximum cell index value (Fig. 3D) raised with the EGF concentration reaching, however, highest values around 3-5 ng/ml of EGF. Thus, IR is a sensitive method to detect in real-time the EGF-induced protrusive activity in a bulk population of cells over a wide range of concentrations.

### IR is a suitable method to monitor protrusion dynamics induced by different growth factors and in different cellular models

One of the drawbacks of IR based techniques is that impedance variations have to be mapped to a known biological behavior. It is therefore of interest to verify whether the analysis based on our 4 quantitative indicators of the curve can be generalized to the use of other factors and cell lines. This largely depends on how universal is the behavior in response to growth factors of different origins and biological functions. To verify this hypothesis we verified the shape and type of response with other cell lines and growth factors.

**Figure 4:**
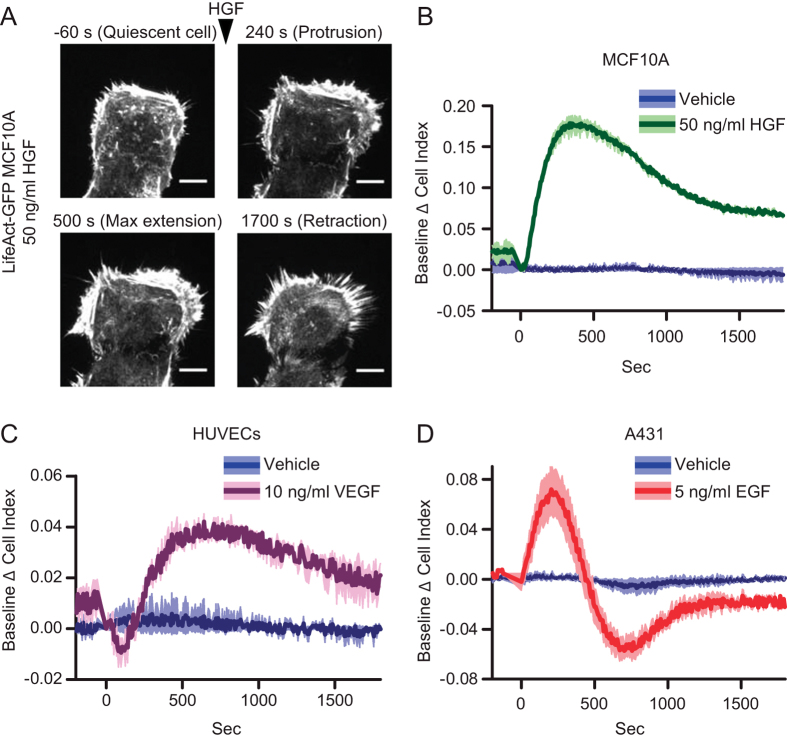
IR of protrusion dynamics in different cellular models and growth factors. (**A**) MCF10A cells were infected with pLKO.1 LifeAct-GFP, deprived of growth factors for 6 hours and kept in a humidified chamber at 37 °C and 5% CO2. Cells were then imaged by means of TIRF microscopy. The depth of the evanescent field was kept at 90 nm. Cells were imaged over a time period of 1780 seconds and stimulated or not with 50 ng/ml HGF. The time at which the stimulus was added was set to t = 0 seconds. Images were acquired every 20 seconds (Video S2). Four time-points are reported in the figure: −60 seconds (before HGF addition, quiescent cell), 240 seconds (protrusion), 500 seconds (maximum extension) and 1700 seconds (retraction). Scale bars, 10 μm. (**B**) Baseline Δ cell index values of MCF10A cells stimulated or not with 50 ng/ml HGF at t = 0. (**C**) Baseline Δ cell index values of HUVECs stimulated or not 10 ng/ml VEGF. (**D**) Baseline Δ cell index values of A431 cells stimulated or not with 5 ng/ml EGF.

First we studied the MCF10A response to the Hepatocyte Growth Factor (HGF) by TIRF microscopy. HGF induced the formation of large protrusions of polymerized actin, visualized by LifeAct-GFP, in form of both lamellipodia and filopodia (Fig. 4A, Video S2). In the same experimental conditions, but monitored by IR, HGF induced the increase of CI, corresponding to the protrusion phase, and the subsequent reduction relative to the retraction phase (Fig. 4B). Next, we evaluated by IR the response of HeLa cells to EGF and HGF stimulation. HeLa cells showed response analogous to that of MCF10A cells with a maximum in the impedance variation followed by retraction at both 5 ng/ml EGF (Fig. S2A) and 50 ng/ml HGF (Fig. S2B).

Furthermore, the IR method was applied to non-epithelial primary cells – human umbilical vein endothelial cells (HUVECs) – stimulated with EGF (Fig. S2C) or Vascular Endothelial Growth Factor (VEGF) (Fig. 4C). Both the growth factors were able to induce a significant increase of impedance, generating curves similar to MCF10A and HeLa cells.

Finally we tested A431 epidermoid carcinoma cells, which harbor Epidermal Growth Factor Receptor (EGFR) overexpression^30^. Both EGF (Fig. 4D) and HGF (Fig. S2D) were able to induce a significant change of impedance, yielding a response qualitatively similar to that obtained with other cell lines and growth factors, showing a rapid protrusion and retraction phases after EGF stimulation. Interestingly the retraction phase did not simply recover the prestimulus values, but showed an overshooting behavior. Furthermore, we noted that HGF stimulation shows slightly different responses regarding the retraction slope and the intensity after stimulus. This suggests that protrusive activity monitored by IR is sensitive to the number of receptors on the cell surface and to the different receptor kinetics. This technique is thus also suitable for quantitative studies on the receptor-ligand dynamics in response to changes in expression, receptor trafficking and temporally varying stimuli.

### The effect of specific drugs on the response can be quantitated by comparing response curve

The suitability of IR for signaling studies was evaluated by inhibiting molecular component of signaling pathways activated by EGF and resulting in protrusion formation. Since the response to signaling is clearly induced by the presence of EGF, we checked whether blocking EGFR would result in absence of response.

**Figure 5:**
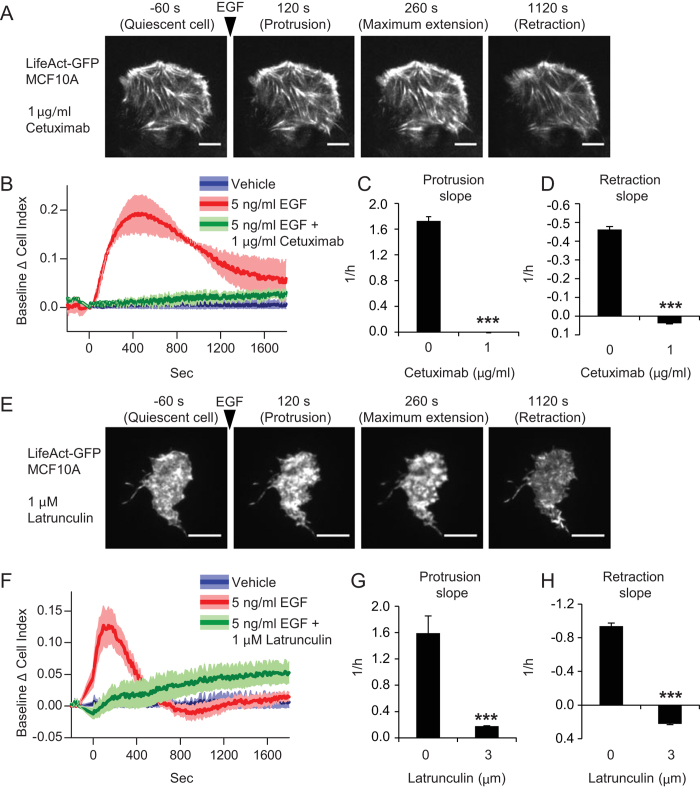
Protrusion is regulated by a signalling pathway starting from EGFR and culminating in actin polymerization. (**A**) MCF10A cells, stably transduced with pLKO.1 LifeAct-GFP, were seeded and maintained in absence of growth factors for 6 hours, pretreated with 1 μg/ml Cetuximab and stimulated with 5 ng/ml EGF at t = 0. Time lapse movie at TIRF microscope of a representative cell (Video S3) was recorded with interval of 20 seconds. Scale bars, 10 μm. (**B**) Baseline Δ cell index values, (**C**) protrusion slope and (**D**) retraction slope of MCF10A treated or not with 1 μg/ml Cetuximab during growth factors deprivation and stimulated with 5 ng/ml EGF, evaluated by IR. (**E**) A representative MCF10A cell, stably transduced with pLKO.1 LifeAct-GFP, was stimulated with 5 ng/ml EGF in presence of 1 μM Latrunculin (Video S4). Scale bars, 10 μm. (**F**) Baseline Δ cell index values, (**G**) protrusion slope and (**H**) retraction slope of MCF10A treated or not with 1 μM Latrunculin during growth factors deprivation, stimulated with 5 ng/ml EGF and evaluated by IR. ***P < 0.001.

Cetuximab is a clinically approved humanized monoclonal antibody that binds and inhibits EGFR^31^. As expected, Cetuximab treatment of MCF10A cells transduced with LifeAct-GFP completely abolished the response in TIRF microscopy experiments (Fig. 5A, Video S3). Analogously, EGF stimulation did not induce a noticeable variation in cell index in Cetuximab-treated cells (Fig. 5B), and both protrusion (Fig. 5C) and retraction slope (Fig. 5D) were significantly reduced compared to untreated cells.

It is well established that EGF acute stimulation activates a signaling cascade that eventually promotes actin polymerization^13^. To confirm this observation we tested whether we could inhibit cell response by blocking actin polymerization with Latrunculin. Latrunculin is a chemical inhibitor that binds to actin preventing its polymerization, thus resulting in a complete disruption of actin cytoskeleton^32^. Treatment of MCF10A with Latrunculin caused a rapid dissolution of stress fibers and prevented protrusion formation (Fig. 5E, Video S4) in TIRF microscopy experiments. The same result was obtained by IR. In presence of Latrunculin the cell index did not increase upon EGF stimulation (Fig. 5F). Thus both protrusion (Fig. 5G) and retraction slope (Fig. 5H) were dramatically reduced.

### Different types of protrusions can be detected by IR

**Figure 6:**
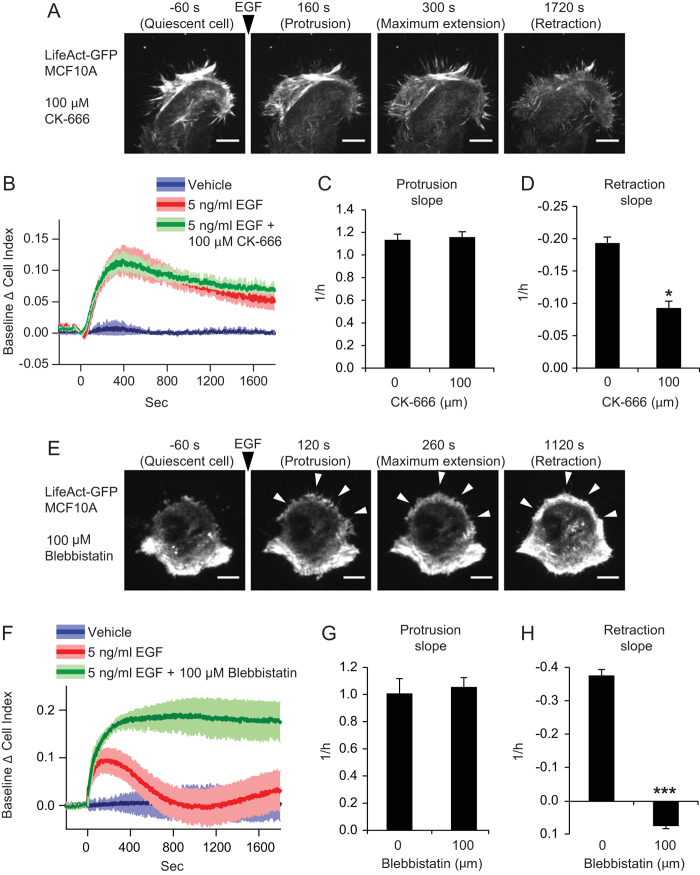
IR of filopodia and the effect of myosin inhibition. (**A**) MCF10A cells, stably transduced with pLKO.1 LifeAct-GFP, were seeded and maintained in absence of growth factors for 6 hours, pretreated with 100 μM CK-666 and stimulated with 5 ng/ml EGF at t = 0. Time lapse movie at TIRF microscope of a representative cell (Video S5) recorded with interval of 20 seconds. Scale bars, 10 μm. (**B**) Baseline Δ cell index values, (**C**) protrusion slope and (**D**) retraction slope of MCF10A treated or not with 100 μM CK-666 during growth factors deprivation, stimulated with 5 ng/ml EGF and evaluated by IR. (**E**) A MCF10A cell, stably transduced with pLKO.1 LifeAct-GFP stimulated with 5 ng/ml EGF in presence of 100 μM Blebbistatin (Video S6). Newly formed lamellipodia are indicated with white arrows. Scale bars, 10 μm. (**F**) Baseline Δ cell index values, (**G**) protrusion slope and (**H**) retraction slope of MCF10A treated or not with 100 μM Blebbistatin during growth factors deprivation, stimulated with 5 ng/ml EGF and evaluated by IR. **P < 0.05, ***P < 0.001.

Cells can form different type of protrusions. Among these, lamellipodia and filopodia are the most extensively characterized in cells grown on 2D. In particular, EGF-stimulated MCF10A cells preferentially produce lamellipodia^29^. However lamellipodia formation can be shifted to filopodia formation by the inhibition of Arp2/3 mediated actin branching^33^,^34^. By treating cells with Arp2/3 inhibitor, we showed that lamellipodia formation in LifeAct-GFP MCF10A stimulated with EGF was completely abolished as shown in Fig. 6A, Video S5. However, observations in TIRF microscopy showed that cells extended filopodia instead of lamellipodia (Fig. 6A, video S5). Therefore, we investigated whether filopodia dynamics was detectable by IR. Cells treated with CK-666 responded to EGF stimulation similarly to untreated cells in terms of cell index (Fig. 6B). Interestingly, no changes in protrusion slope were detected (Fig. 6C), while we noticed a moderate decrease of retraction slope (Fig. 6D). These data indicate that retraction kinetics in filopodia might be regulated differently than in lamellipodia and provide evidence that this technique is able to detect adhesive protrusions of different sizes and types. In addition, these results demonstrate the advantages of comparing direct TIRF microscopy observations with IR measurements.

### IR reveals the myosin role in the retraction phase of protrusion dynamics

Although it has been shown that lamellipodia have continuous cycles of protrusion and retraction^12^, retraction is much less characterized than protrusion, in particular for what concerns molecules and signaling pathways involved. For instance, it is unclear if the speed of retraction is directly dependent on the tension of the plasma membrane or whether a myosin-dependent active contraction is involved^35^,^36^. We therefore investigated if the retraction phase in our model was dependent on myosin activity, treating MCF10A cell with Blebbistatin, an inhibitor of myosin-II ATPase activity^37^. As expected, the presence of Blebbistatin did not impair EGF-induced lamellipodia extension, which is known to be a process that does not require myosin activity. However, lamellipodia were not retracted after their formation and instead were maintained extended during the observation period (Fig. 6E, Video S6). Similarly, Blebbistatin treatment on MCF10A induced a sharp increase of cell index monitored by IR after EGF stimulation. However, after having reached the maximum value, cell index values remained stationary (Fig. 6F). Consequently, treatment with Blebbistatin did not reduce protrusion slope (Fig. 6G), while completely abolished the retraction slope (Fig. 6H). These data indicate that retraction phase monitored in real-time by IR is a process mediated by myosin contraction and seem to rule out actin de-polymerization as the sole responsible mechanism for protrusion retraction.

## Discussion

Lamellipodia, together with filopodia, are the most frequently observed protrusive structures in cells migrating in a 2D environment^1^. Here we report quantitative measurements of protruding activity of epithelial cells upon growth factor stimulation by means of IR techniques. By directly comparing TIRF microscopy experiments and IR measurements we were able to dissect the impedance response into distinct phases that can be separately altered by specific drugs. Indeed, formation and retraction of cell protrusions exactly coincide with the increase and decrease of IR signal, respectively. The dynamics and extent of the process can be precisely quantified and it is analogous to that detected by TIRF microscopy or reported in literature^13^,^38^,^39^.

The IR method is effective to monitor protrusive activity induced by several pro-migratory factors, such as EGF, HGF and VEGF, and is applicable to different types of cells. Furthermore, this method is suitable for functional study of signaling pathway involved in protrusive activity and, potentially, in cell migration. For example, an EGFR blocking antibody completely inhibited the effects of EGF on protrusion formation.

EGF was shown to activate the small GTPases Rac1 and Cdc42 at the leading edge^40^ and Rac1 activation was shown to be downstream of MAPK pathway upon EGF stimulation in the formation of lamellipodia^13^. The ultimate effect of these signaling cascades is the activation of actin polymerization at the front of growing lamellipodia or filopodia. Our results confirm that impedance variations detected after EGF stimulation are indeed totally dependent on actin polymerization, in agreement with published evidence^1^. Interestingly, thanks to IR we are able to detect not only lamellipodia but also filopodia, as shown in cells treated with an Arp2/3 inhibitor.

What molecular players act in the phase of retraction of the lamellipodium is more debated. Although lamellipodia do not contain myosin^41^, it has been reported that at the peak of protrusion myosin II filaments form in lamella, a more stable region of cellular protrusions, driving the formation of actin-arc and then shrinking protrusions^12^. Our results indicate that, upon growth factors induced protrusion, retraction phase is determined by myosin contraction. This suggests that myosin activity might be important not only in the cell tail retraction but also in the leading edge dynamics. A critical role for myosin in periodic contractions at the leading edge has been reported, where myosin II pulls the rear of the lamellipodial actin network, causing upward bending, edge retraction, and initiation of new adhesion sites^42^. Here we showed that after stimulation with growth factors, similar lamellipodia dynamics are observable. It is worth noting that protrusion and contraction are synchronized in the population due to the starving, thus making the response particularly clean and reproducible.

The response to a soluble cue, such as a growth factor, is particularly relevant during directed migration of mesenchymal and epithelial cells. How lamellipodia dynamics contribute to directional cell migration is not completely known, however very recently it has been shown that localized Myosin II inactivation provides the asymmetry of force needed for directional migration of mesenchymal cells^43^.

Interestingly, it has been shown that lamellipodia are critical protrusive structures also in 3D migration^2^,^4^. A recent paper^44^ introduces the possibility of integrating microfluidics with impedance reading to measure chemotaxis in 3D matrices. Although this kind of application of impedance based assays are presently limited by the need of cell-electrode contact, we cannot exclude interesting future progress in this field.

In conclusion we have presented quantitative measurements on protrusion dynamics that map distinct response phases as observed in TIRF microscopy to separate regimes of impedance variation in IR measurements. Such clear and direct correspondence makes the interpretation of drug treatments and genetic modifications straightforward. Furthermore, we present data on how the retraction of the lamellipodium produced upon acute growth factor stimulation is driven by myosin and rule out the possibility that this is solely driven by actin de-polymerization.

## Materials and methods

### Cell lines

MCF10A (CRL-10317), 293T (CRL-11268), A431 (CRL-1555) and HeLa (CCL-2) cell lines were obtained from ATCC resource center ( http://www.atcc.org). All experiments were performed on cell lines passaged for <3 months after thawing. 293T, A431 and HeLa cells were cultured in Dulbecco’s Modified Eagle’s Medium, DMEM, (Sigma-Aldrich, St Louis, MO, USA). The culture medium was supplemented with 10% fetal bovine serum (Gibco, Life Technologies, Rockville, MD, USA), 200 U/ml of penicillin and 200 μg/ml streptomycin (Sigma-Aldrich, St Louis, MO, USA). MCF10A cells were cultured as described^45^. Human EC were isolated from umbilical cord veins, characterized and grown in M199 (Sigma-Aldrich, St Louis, MO, USA) containing 20% fetal bovine serum (Gibco, Life Technologies, Rockville, MD, USA), bovine brain extract, heparin (50 μg/ml, Sigma-Aldrich, St Louis, MO, USA) and penicillin-streptomycin (200 U/ml, Sigma-Aldrich, St Louis, MO, USA) on gelatin-coated tissue culture dishes, as previously described^46^. Human umbilical cords were kindly donated by O.I.R.M- S. Anna Hospital (Agreement n.619, 19/06/07) with prior written informed consent.

### Reagents

Human Epidermal Growth Factor (EGF) and Vascular Endothelial Growth Factor (VEGF)-A165 were purchased from R&D Systems, Minneapolis, MN, USA. Human Hepatocyte Growth Factor (HGF) was purchased from PeproTech, Rocky Hill, NJ, USA. Cetuximab was purchased from Merck KGaA, Darmstadt, Germany. Latrunculin A and CK-666 from Sigma-Aldrich, St Louis, MO, USA. Blebbistatin from Calbiochem, San Diego, CA, USA.

### Impedance reading (IR) of protrusion dynamics

IR is a label-free non-invasive technique based on the measurement of the frequency dependent electrical impedance of cell-covered electrodes subject to an alternate small electric current. This method was originally invented and developed by Giaever and Keese^20^,^21^. IR methods have been successfully applied to the study of cell adhesion^22^,^47^,^48^, cell proliferation and viability^49^, cell migration^50^, trans-endothelial invasion^51^, wound healing^52^, cell-cell adhesion^26^, apoptosis^24^ and cytotoxicity^25^.

The theoretical details of how this method works are detailed elsewhere^21^, here we only give a succinct description of the experimental system. Briefly cells adhering on top of the electrode can vary the impedance of the electrode by increasing the measured voltage drop either influencing paracellular (avoiding the cell) or transcellular (passing through the cell) currents. The relative importance of these two contributions depends on the frequency of the applied current. In the settings of the commercial system we are using (xCELLigence), the impedance is mostly due to transcellular currents. Indeed the impedance values obtained at intermediate frequencies (in the 1 to 10 kHz range) depend both on the fraction of the electrode area covered with the spreading cells and on the space between the electrode and the basal cell membrane. On the contrary at higher frequencies (in the 10 to 50 kHz range) the capacitive contribution to the impedance is dominating. Thus in this regime the measurements of impedance reflect essentially the fraction of the electrode covered with cells, thereby being a large scale substitute for area measurements in microscopy experiments^53^.

There exist a number of different applications to this method in cell biology and several commercially available setups including ECIS (Electric Cell-substrate Impedance Sensing) from Applied Biophysics, xCELLigence (Real Time Cell Electric Sensing; RT-CES) from Acea Biosciences, the one used in this paper, and Cellkey (Cellular Dielectric Spectroscopy; CDS) from Molecular Devices.

Experiments were performed using the xCELLigence RTCA DP instrument (ACEA, San Diego, CA, USA) which was placed in a humidified incubator at 37 °C and 5% CO2. IR of protrusion dynamics was performed using modified 16-well plates with micro electrodes attached at the bottom of the wells (E-plate, ACEA, San Diego, CA, USA). Cells were seeded one day before the assay in culture medium. The following day complete medium was substituted with 100 μl/well of culture medium without growth (DMEM supplemented 200 U/ml of penicillin and 200 μg/ml streptomycin). After 6 hours of EGF deprivation, the E-plates were placed on the RTCA DP instrument and the impedance value of each well was automatically acquired by the xCELLigence system and expressed as a Cell Index value. Impedance was recorded every 5 seconds for one hour. At least two technical replicates of each experimental condition were used in each biological replicate. After 2 minutes, growth factors were added with an additional volume of 100 μl/well. Inhibitors were added one hour before growth factors addition during to the EGF deprivation period and then added together with growth factors to maintain the same final concentrations. In order to correctly take into account the baseline value of impedance, the value of the first time-point after EGF addition was subtracted to the cell index values. Furthermore, point-by-point subtraction of the values of unstimulated cells was performed on each curve, to obtain the Baseline Δ Cell index values. The Slope of Baseline Δ Cell index curves in a chosen Time period was calculated by fitting the points to a straight line. The protrusion slope is calculated as the mean slope between t^0^ and t^m^, where t^0^ is the first time point after growth factor addition and t^m^ is the time point at which Baseline Δ Cell index reaches the maximum value. The retraction slope is calculated as the mean slope between t^m^ and t^2m^, where t^2m^ is twice t^m^.

### Lentivirus production

LifeAct-GFP was kindly provided by Roland Wedlich-Söldner, Max Planck Institute of Biochemistry, Martinsried, Germany, and was inserted in pLKO.1 lentiviral vector in the place of puromicin resistance sequence as previously described^54^. Lentivirus were produced by calcium phosphate transfection of lentiviral plasmids (pLKO.1 LifeAct-GFP) together with packaging (pCMVdR8.74) and envelope (pMD2.G-VSVG) plasmids in 293T cells as previously described^55^. Supernatant was harvested 24 and 48 hours post-transfection, filtered with 0.45 μm filters, precipitated (19000xG for 2 hours at 20 °C) and suspended in PBS at a higher concentration. The multiplicity of infection (MOI) was determined by infecting HeLa cells in presence of 8 μg/ml of polybrene and the quantification of GFP-positive cells was performed by flow cytometry.

### LifeAct-GFP dynamics with TIRF microscopy

MCF10A cells were infected with pLKO.1 LifeAct-GFP using a M.O.I. equal to 2. Then cells were seeded at low density on glass bottom plates (Porvair, Norfolk, UK) coated with 1 μg/ml fibronectin (Sigma-Aldrich, St Louis, MO, USA). After an overnight starving in medium w/o growth factors (DMEM supplemented 200 U/ml of penicillin and 200 μg/ml streptomycin), cells were placed on an inverted microscope equipped with a 37 °C humidified chamber with 5% CO2, and visualized using True MultiColor Laser TIRF Leica AM TIRF MC (Leica Microsystems, Wetzlar, Germany) equipped with a 63X oil immersion objective (HCX PL APO 63x/1.47 OIL CORR TIRF) and Hamamatsu EM-CCD camera C9100-02. Images were acquired through Leica LAS AF6000 modular system software. The depth of the evanescent field was kept at 90 nm. Time lapse movies were performed with a 20 seconds interval and EGF or HGF were added at the 4^th^ frame. Cell area was calculated using ImageJ^56^.

### Statistical analysis

For scatter plot representation, the central line depicts median values. All the remaining data are represented as mean value of both technical and biological replicates, while error bars report the standard deviation. Statistical significance was determined by Student’s t-test.

## Author Contributions

P.A.G., A.P. and L.P. conceived the idea and wrote the manuscript; P.A.G., A.P. and F.C. performed the experiments and analyzed the data; L.d.B., D.S., G.S. and F.B. analyzed and discussed the data; all authors reviewed and approved the manuscript.

## Additional Information

**How to cite this article**: Armando Gagliardi, P. *et al*. Real-time monitoring of cell protrusion dynamics by impedance responses. *Sci. Rep.* **5**, 10206; doi: 10.1038/srep10206 (2015).

## Supplementary Material

Supplementary Information

Supplementary Video 1

Supplementary Video 2

Supplementary Video 3

Supplementary Video 4

Supplementary Video 5

Supplementary Video 6
