# Supplementary material for: Real-time monitoring of cell protrusion dynamics by impedance responses
Source: Sci Rep. 2015 May 15;5:10206. doi: 10.1038/srep10206 (PMC4432390; doi:10.1038/srep10206)
Supplement: Supplementary Information [file srep10206-s1.pdf]

Supplementary information for

Real-time monitoring of cell protrusion dynamics by impedance responses

Paolo Armando Gagliardi[1,2], Alberto Puliafito[2], Laura di Blasio[1,2], Federica Chianale[2,3], Desiana Somale[1,2], Giorgio Seano[1,2,4], Federico Bussolino[1,2,5] and Luca Primo[1,2,5]

1. Department of Oncology, University of Torino, Turin, 10060, Italy
2. Candiolo Cancer Institute-FPO IRCCS, Candiolo, 10060, Italy
3. Current address: Bracco Imaging SpA, Colleretto Giacosa (TO), 10010, Italy
4. Current address: Edwin Steele Laboratory for Tumor Biology, Department of Radiation Oncology, Harvard Medical School, Massachusetts General Hospital, Boston, 02114, USA
5. Center for Molecular Systems Biology, 10124, Torino, Italy

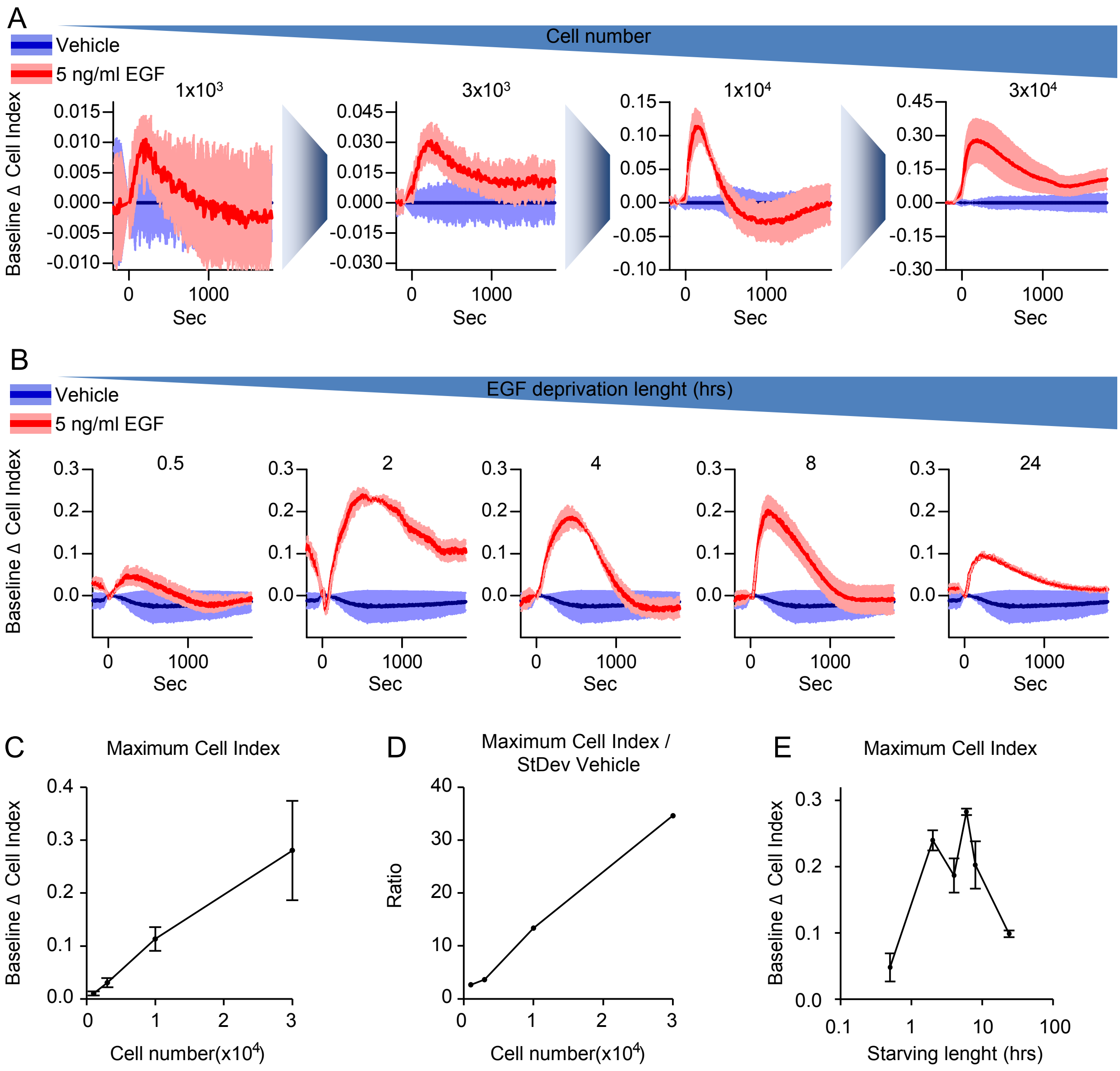

Figure S1. **IR responses are influenced by cell density and EGF deprivation length.** (A) IR responses of MCF10A cells seeded with different densities: $1x10^3$, $3x10^3$, $1x10^4$, $3x10^4$ cells/well and stimulated with 5 ng/ml EGF. (B) IR responses of MCF10A after EGF deprivation for 0.5, 2, 4, 8, 24 hours and then stimulated with 5 ng/ml EGF. (C) Maximum values of Baseline Δ cell index of different densities of MCF10A cells deprived of EGF for 6 hours and stimulated with 5 ng/ml EGF. (D) Ratio between maximum values of EGF stimulated curves and standard deviation of vehicle in MCF10A cells seeded at different cell densities and stimulated with 5 ng/ml EGF. (E) Maximum values of Baseline Δ cell index curves of $5x10^3$ MCF10A cells deprived of EGF for different intervals and stimulated with 5 ng/ml EGF.

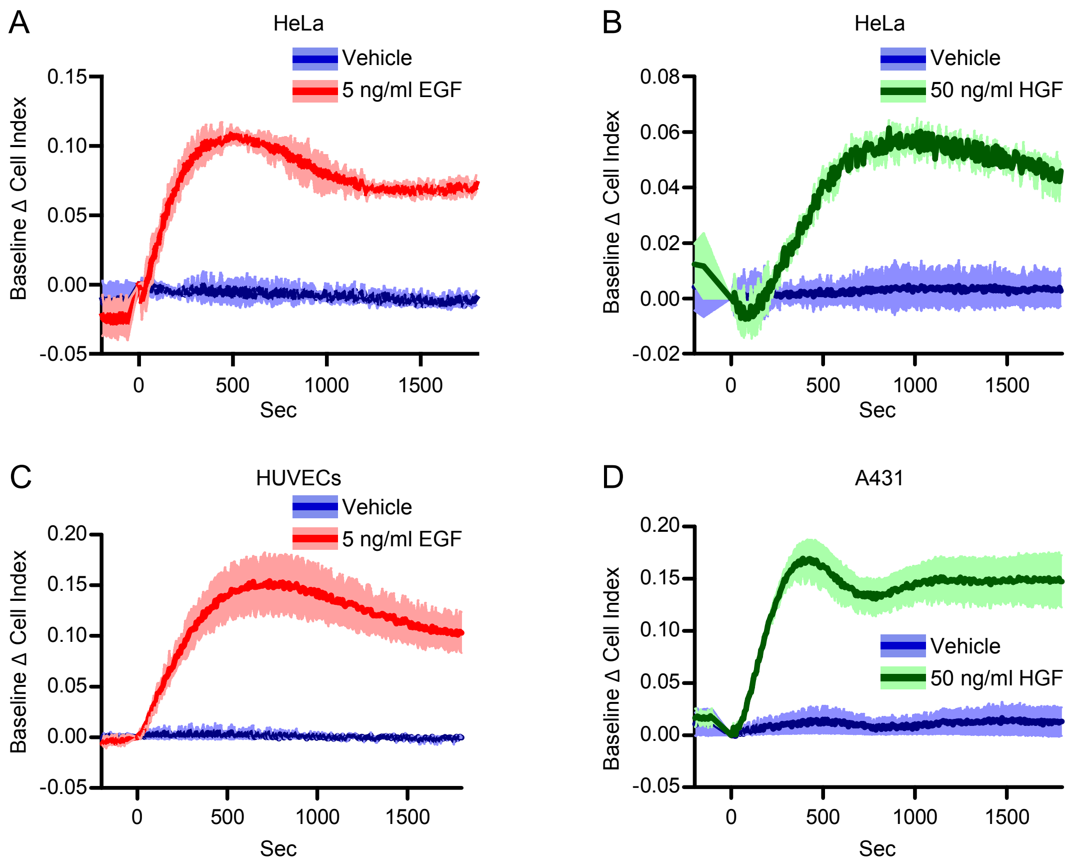

Figure S2. **IR of protrusion dynamics in different cellular models and growth factors.** (A) Baseline Δ cell index values of HeLa cells stimulated or not with 5 ng/ml EGF or (B) 50 ng/ml HGF. (C) Baseline Δ cell index values of HUVECs stimulated or not with 5 ng/ml EGF. (D) Baseline Δ cell index values of A431 cells stimulated or not with 50 ng/ml HGF.

Video legends

Video S1. EGF induces lamellipodia protrusion and retraction in MCF10A cells. MCF10A cells were infected with pLKO.1 LifeAct-GFP, deprived of EGF for 6 hours and kept in a humidified chamber at 37°C and 5% CO2. Cells were then imaged by means of TIRF microscopy. The depth of the evanescent field was kept at 90 nm. Cells were imaged over a time period of 1180 seconds and stimulated or not with 5 ng/ml EGF. The time at which the stimulus was added was set to t = 0 seconds. Images were acquired every 20 seconds.

Video S2. HGF induces lamellipodia protrusion and retraction in MCF10A cells. MCF10A cells were infected with pLKO.1 LifeAct-GFP, deprived of growth factors for 6 hours and kept in a humidified chamber at 37°C and 5% CO2. Cells were then imaged by means of TIRF microscopy. The depth of the evanescent field was kept at 90 nm. Cells were imaged over a time period of 1780 seconds and stimulated or not with 50 ng/ml HGF. The time at which the stimulus was added was set to t = 0 seconds. Images were acquired every 20 seconds.

Video S3. EGF signalling regulating lamellipodia protrusion and retraction are transduced by EGFR. MCF10A cells were infected with pLKO.1 LifeAct-GFP, deprived of EGF for 6 hours and kept in a humidified chamber at 37°C and 5% CO2. Cells were then imaged by means of TIRF microscopy. The depth of the evanescent field was kept at 90 nm. One hour before EGF stimulation cells were treated with 1 µg/ml Cetuximab, which was then maintained for all the duration of the assay. Cells were imaged over a time period of 1180 seconds and stimulated or not with 5 ng/ml EGF. The time at which the stimulus was added was set to t = 0 seconds. Images were acquired every 20 seconds.

Video S4. EGF mediated lamellipodia protrusion is executed by actin polymerization. MCF10A cells were infected with pLKO.1 LifeAct-GFP, deprived of EGF for 6 hours and kept in a humidified chamber at 37°C and 5% CO2. Cells were then imaged by means of TIRF microscopy. The depth of the evanescent field was kept at 90 nm. One hour before EGF stimulation cells were treated with 1 µM Latrunculin, which was then maintained for all the duration of the assay. Cells were imaged over a time period of 1180 seconds and

stimulated or not with 5 ng/ml EGF. The time at which the stimulus was added was set to t = 0 seconds. Images were acquired every 20 seconds.

Video S5. IR detects filopodia dynamics. MCF10A cells were infected with pLKO.1 LifeAct-GFP, deprived of EGF for 6 hours and kept in a humidified chamber at 37°C and 5% CO2. Cells were then imaged by means of TIRF microscopy. The depth of the evanescent field was kept at 90 nm. One hour before EGF stimulation cells were treated with 100 µM CK-666, which was then maintained for all the duration of the assay. Cells were imaged over a time period of 1780 seconds and stimulated or not with 5 ng/ml EGF. The time at which the stimulus was added was set to t = 0 seconds. Images were acquired every 20 seconds.

Video S6. Myosin contraction is required for lamellipodia retraction. MCF10A cells were infected with pLKO.1 LifeAct-GFP, deprived of EGF for 6 hours and kept in a humidified chamber at 37°C and 5% CO2. Cells were then imaged by means of TIRF microscopy. The depth of the evanescent field was kept at 90 nm. One hour before EGF stimulation cells were treated with 100 µM Blebbistatin, which was then maintained for all the duration of the assay. Cells were imaged over a time period of 1180 seconds and stimulated or not with 5 ng/ml EGF. The time at which the stimulus was added was set to t = 0 seconds. Images were acquired every 20 seconds.
